# Influence of Indoleamine-2,3-Dioxygenase and Its Metabolite Kynurenine on γδ T Cell Cytotoxicity against Ductal Pancreatic Adenocarcinoma Cells

**DOI:** 10.3390/cells9051140

**Published:** 2020-05-06

**Authors:** Hannah Jonescheit, Hans-Heinrich Oberg, Daniel Gonnermann, Martin Hermes, Vjola Sulaj, Christian Peters, Dieter Kabelitz, Daniela Wesch

**Affiliations:** Institute of Immunology, University Hospital Schleswig-Holstein Campus Kiel, D-24105 Kiel, Germany; hannah.jonescheit@gmail.com (H.J.); hans-heinrich.oberg@uksh.de (H.-H.O.); d.gonnermann@gmx.de (D.G.); mail@martinhermes.de (M.H.); vjola_s@hotmail.com (V.S.); Christian.Peters@uksh.de (C.P.); Dietrich.Kabelitz@uksh.de (D.K.)

**Keywords:** gamma delta T cells, ductal pancreatic adenocarcinoma, indoleamine-2,3-dioxygenase, kynurenine, cytotoxicity, bispecific antibody

## Abstract

Background: Pancreatic ductal adenocarcinoma (PDAC) is a malignant gastrointestinal disease. The enzyme indoleamine-2,3-dioxgenase (IDO) is often overexpressed in PDAC and its downstream metabolite kynurenine has been reported to inhibit T cell activation and proliferation. Since γδ T cells are of high interest for T cell-based immunotherapy against PDAC, we studied the impact of IDO and kynurenine on γδ T cell cytotoxicity against PDAC cells. Methods: IDO expression was determined in PDAC cells by flow cytometry and Western blot analysis. PDAC cells were cocultured with γδ T cells in medium or were stimulated with phosphorylated antigens or bispecific antibody in the presence or absence of IDO inhibitors. Additionally, γδ T cells were treated with recombinant kynurenine. Read-out assays included degranulation, cytotoxicity and cytokine measurement as well as cell cycle analysis. Results: Since IDO overexpression was variable in PDAC, IDO inhibitors improved γδ T cell cytotoxicity only against some but not all PDAC cells. γδ T cell degranulation and cytotoxicity were significantly decreased after their treatment with recombinant kynurenine. Conclusions: Bispecific antibody drastically enhanced γδ T cell cytotoxicity against all PDAC cells, which can be further enhanced by IDO inhibitors against several PDAC cells, suggesting a striking heterogeneity in PDAC escape mechanisms towards γδ T cell-mediated anti-tumor response.

## 1. Introduction

Pancreatic ductal adenocarcinoma (PDAC) is among the most common pancreatic tumors and, with an extremely aggressive malignancy, is the 4th leading cause of cancer-related death. Since the disease is usually diagnosed at an advanced stage of the disease, surgical treatment options are often limited at the time of diagnosis. Resistance to chemotherapy and radiotherapy is frequently observed, impairing the remaining life expectancy of patients [1,2,3]. The overall 5 year survival rate of pancreatic cancer patients is approximately 8% [4]. A characteristic feature of PDAC is the inflammatory and immunosuppressive microenvironment of the tumor which contributes significantly to the progression and manifestation of the tumor and which display a high heterogeneity within tumor cells [5]. The desmoplastic stroma surrounding PDAC tumors compromises both a non-cellular (extracellular matrix) and a cellular compartment composed of diverse (immunosuppressive) cells such as tumor-associated macrophages (TAMs), myeloid-derived suppressor cells (MDSCs), mesenchymal cells (e.g., fibroblasts) and regulatory T cells (Treg), preventing the penetration of cytotoxic T lymphocytes to the tumor site [5,6]. An immunosuppressive tumor microenvironment also hinders γδ T cells from exerting their cytotoxic activity or may promote the differentiation of γδ T cell subsets to an immunosuppressive phenotype due to their high plasticity [7,8]. Human γδ T cells can be classified in at least three major T cell subsets as follows (i) Vγ9Vδ2 γδ T cells constitute the main population in the peripheral blood, (ii) Vδ1 γδ T cells are mainly located in the skin and mucosa, and (iii) Vδ3 γδ T cells are present in the liver. Human Vδ2 γδ T cells recognize T cell receptor (TCR) ligands F1-ATPase in complex with apolipoprotein A-I, DNA mismatch repair protein MSH2, and pyrophosphate intermediates of a dysregulated mevalonate pathway of tumor cells or the non-mevalonate pathway in bacteria. While the ligands for Vδ3 γδ T cells are so far not determined, Vδ1 γδ T cells recognize microbial and self-lipids bound to non-classical CD1d molecules and stress-induced MHC class-I related chain A/B (MICA/B) [8,9]. All human γδ T cell subsets can infiltrate into tumor tissue [10,11,12,13,14]. Bioinformatic analyses of meta-genomic datasets determined the relative abundance of γδ T cells within different tumor entities and correlated this with patient outcome [15]. Recently, we and others demonstrated that γδ T cells infiltrate into PDAC tissue [10,16,17]. Although the number of Vδ1 γδ T cells is higher compared to Vδ2 γδ T cells in PDAC tissue, Vδ2 γδ T cells are still present [10]. Vγ9Vδ2 γδ T lymphocytes are a very interesting effector cell population for T cell-based immunotherapy due to their HLA-unrestricted recognition of phosphorylated antigens (PAg), their ability to present antigens and their reduced potential to cause graft versus host disease [9,18].

An impaired Vγ9Vδ2 γδ T cell cytotoxicity can be modulated by T cell engagers. Chimeric antigen receptor-engineered T cell constructs as well as bispecific antibodies (bsAb) are of interest in cancer immunotherapy for recruitment of effector cells to the tumor site [19,20,21]. Regarding bsAb, tribody [(HER2)_2_×Vγ9] with a specificity for human epidermal growth factor receptor (HER)-2 expressed on PDAC cells and Vγ9 as part of the Vγ9Vδ2 γδ TCR selectively targets γδ T cells to PDAC cells and enhances their cytotoxicity mediated by an increased release of cytotoxic granules [16,22]. The enhancement of T cell cytotoxicity complies with the current purposes of T cell-based immune therapies and/or targeted therapies to stave off immunosuppressive mechanisms. The heterogeneity within PDAC cells as well as the diverse interactions with tumor-infiltrating cells or stromal cells and the selective pressure in this tumor microenvironment by, e.g., oxidative and metabolic stress, requires profound knowledge about the different tumor escape mechanisms to improve the outcome by the application of combined therapies.

The existence of different intrinsic tumor escape mechanisms targeting several but not all PDAC cells further underlines the heterogeneity within PDAC cells. For instance, an upregulation of the adhesion molecule L1CAM (CD171) by several PDAC cells is associated with an epithelial mesenchymal transition and an upregulation of mesenchymal protein vimentin, which correlates with a higher tumor grade, and can influence Treg migration [23,24]. The overexpression of tumor necrosis factor (TNF)-related apoptosis inducing ligand (TRAIL) receptor 4 mainly induces pro-tumoral and anti-apoptotic functions in PDAC cells [25,26]. Unexpectedly, an enhanced TRAIL-R4 expression induced the cytotoxic activity of γδ T cells against some PDAC cells [27]. The release of tumor necrosis factor (TNF)-α by activated γδ T cells increased the prostaglandin (PG) synthetase cyclooxygenase (COX)-2 expression in a few PDAC cells, and thereby induced the massive release of PGE_2_ that inhibits γδ T cell cytotoxicity. This inhibition was reverted by the application of a COX-2 inhibitor or by knock-in of TRAIL-R4 together with tribody [(HER2)_2_×Vγ9] or bispecific single chain fragment variable (bsscFv) [HER2xCD3] [27,28].

In addition, a further intrinsic tumor escape mechanism by which PDAC cells evade the immune system relates to changes in the metabolism of the essential amino acid tryptophan. Degradation of tryptophan by the enzyme indoleamine-2,3-dioxygenase (IDO)-1 represents an important anti-proliferative strategy of the cellular immune response [29,30,31]. IDO-1 and -2 as well as tryptophan 2,3-dioxygenase (TDO) are enzymes that are expressed in moderate concentrations in all tissues and convert tryptophan into kynurenine [32,33,34]. A further metabolite of this pathway (more downstream) is picolinic acid, which is suggested to stimulate macrophages [33,35]. An increased IDO-1 production, mediated by, e.g., inflammatory conditions in response to T helper-1 cytokine interferon (IFN)-γ, protects healthy tissue in areas in which T cells are massively activated, e.g., in the tumor microenvironment [36,37,38]. However, an increased IDO production of tumors themselves is suggested as a key regulator of T cell immune responses [39,40,41,42,43]. Low tryptophan levels lead to cell cycle arrest and T lymphocyte apoptosis by activating the general control non-derepressible (GCN)-2 kinase [44]. The accumulation of the tryptophan metabolites in the micromolar range detected in tumors leads to the differentiation of a regulatory phenotype in CD4 T cells via binding to the aryl hydrocarbon receptor (AHR) and a reduction in the T cell cytotoxicity [45,46,47]. Further, the expression of IDO in tumors also appears to be related to oncogenic signaling pathways that increase the expression by activating transcription factors such as signal transducer and activator of transcription (STAT)-3 or nuclear factor kappa-light-chain enhancer of activated B cells (NF- κB) [48,49]. Drugs targeting IDO and TDO pathways are already applied in clinical trials with the intention to reverse cancer-induced immunosuppression [50,51,52,53].

In this study, we focused on IDO-1 and -2, which are described to be overexpressed in some pancreatic cancer cells [54,55], and their influence on Vγ9Vδ2 γδ T cell cytotoxicity against PDAC cells and their consequences for their application in immunotherapy.

## 2. Materials and Methods

### 2.1. Generation of Short-Term Activated Vγ9Vδ2 γδ T Cells

Leukocyte concentrates from healthy adult blood donors were kindly provided by the Department of Transfusion Medicine of the University Hospital Schleswig-Holstein (UKSH) in Kiel, Germany. In addition, heparinized blood from PDAC patients was obtained from the Department of General and Thoracic Surgery (UKSH, Campus Kiel) and from the Surgery Department of the Community Hospital in Kiel distributed by the Biobank BMB-CC of the PopGen 2.0 Biobanking Network (P2N; UKSH, Campus Kiel) supervised by Dr. C. Röder and Prof. Dr. S. Sebens (Institute for Experimental Cancer Research, Kiel, Germany). In accordance with the Declaration of Helsinki, written informed consent was obtained from all donors, and the research was approved by the relevant institutional review boards (ethic committee of the Medical Faculty of the CAU Kiel, code number: D 405/10, D445/18 and A110/99). Peripheral blood mononuclear cells (PBMCs) were isolated from leukocyte concentrates or heparinized blood from PDAC patients by Ficoll-Hypaque (Biochrom, Berlin, Germany) density gradient centrifugation. Cells were cultured in RPMI 1640 supplemented with 25 mM HEPES, 2 mM l-glutamine, 100 µg/mL streptomycin, 100 U/mL penicillin and 10% fetal bovine serum (FBS, Thermo Fisher Scientific, Karlsruhe, Germany) [complete medium]. Short-term activated Vγ9Vδ2 γδ T cells were expanded by stimulation of PBMCs with 300 nM phosphoantigen (PAg) bromohydrinpyrophophate (BrHPP, Innate Pharma, Marseille, France), which induces a selective outgrowth of Vγ9Vδ2-expressing γδ T cells. Since resting, initially stimulated Vγ9Vδ2 γδ T cells produce low amounts of IL-2, 50 IU/mL rIL-2 were added every two days over a culture period of 14 days. After two weeks, Vγ9Vδ2 γδ T cells had a purity of 80%–99%. The x-fold increase in the selectively expanded Vγ9Vδ2 γδ T cells was between 100 and 1000. Short-term activated Vγ9Vδ2 T cells with a purity < 95% were labeled with anti-TCRαβ mAb clone IP26 (BioLegend, San Diego, CA, USA) and subjected to magnetic separation in order to deplete remaining αβ T cells.

### 2.2. Tumor Cell Lines

Human PDAC cell lines BxPC3, Panc-1, PancTu-I and Capan-2 derived from primary tumors (stage G1–G3), Panc89 and Colo357 cells established from lymph node metastases (stage G1–G2) as well as Capan-1 cells generated from liver metastases (stage G1) were cultured in complete medium under regular conditions (5% CO_2_, humidified, 37 °C). The PDAC cell lines were kindly provided by Dr. C. Röder, Prof. Dr. A. Trauzold and Prof. Dr. S. Sebens, Institute for Experimental Cancer Research, Kiel, Germany. Human breast cancer cell line MCF-7 (ATCC, Manassas, VA, USA) was also cultured in complete medium. For detachment of adherent tumor cells, 0.05% trypsin/0.02% EDTA (Biochrom) was used. Genotyping of tumor cells was carried out by short tandem repeat analysis and absence of mycoplasma was routinely confirmed by RT-PCR (Venor^®^ GEM classic, Minerva Biolabs GmbH, Germany).

### 2.3. Flow Cytometry

For analysis of purity, 2 × 10^5^ Vγ9Vδ2 γδ T cells were washed and stained with mAb as follows: anti-CD3 (clone SK7, BD Biosciences, Heidelberg, Germany), anti-TCRγδ (clone 11F2, Miltenyi Biotec, Bergisch Gladbach, Germany), anti-TCRαβ (clone IP26, BioLegend), anti-TCRVδ2 (clone Immu389, Beckman Coulter, Krefeld, Germany), anti-TCRVγ9 (clone 7A5 [56]), and corresponding isotype controls (BD Biosciences or BioLegend) for 25 min. After washing, cells were analyzed by flow cytometry (LSR-Fortessa, BD Biosciences) using Diva 8 or FlowJo software.

For surface staining of PDAC cells, 2 × 10^5^ cells were washed and treated with 20 µL of a 1:20 diluted Fc-blocking reagent (Miltenyi Biotec) for 15 min. After a washing step, cells were stained with PE-Vio770-conjugated anti-HER-2 clone 24D2 mAb (Miltenyi Biotec) or appropriate isotype control and analyzed on a flow cytometer. For intracellular staining, 2 × 10^5^ tumor cells were permeabilized and fixed with Cytofix/Cytoperm kit (BD Biosciences) and stained with 10 μg/mL anti-pan-IDO-APC mAb (clone #700838, R&D Systems, Wiesbaden, Germany) or corresponding isotype controls. After washing, all samples were analyzed by flow cytometer (FACS Calibur Analyzer, BD Biosciences) using CellQuestPro or FlowJo software.

### 2.4. Western Blot Analysis

In total, 10^6^ PDAC cells were cultured in medium or treated with 100 ng/mL or 500 ng/mL rIFN-γ (R&D Systems) for 48 h. Thereafter, cells were lysed in TNE lysis buffer with 1% (*v*/*v*) Nonidet P-40 (Fluka Chemie, Buchs, Switzerland) in 50 mM Tris (Roth, Karlsruhe, Germany), 2 mM EDTA, 150 mM NaCl (Merck, Darmstadt, Karlsruhe), with phosphatase and protease inhibitors sodium fluoride and sodium orthovanadate (both from Merck) and the complete protease inhibitor mix^TM^ (Roche, Mannheim, Germany). Protein concentration was determined by Coomassie (Thermo Fisher Scientific) using the Bradford method. In total, 10 μg protein was separated by SDS-PAGE (Merck), blotted on a nitrocellulose membrane (Hybond C-Extra, GE Healthcare, Munich, Germany), probed with anti-IDO-1 mAb (clone 1A3, OriGene Technologies, Herford, Germany; 0.5 μg/mL) or IDO-2 mAb (clone 1A4, OriGene Technologies, 0.5 μg/mL), and detected by peroxidase (POD)-conjugated sheep anti-mouse mAb (1:7500). In total, 0.22 mg/mL rIDO (R&D Systems) was used as a control. For analysis of β-actin as loading control, peroxidase was inactivated with 15% H_2_O_2_ and the membrane was reprobed with anti-β-actin mAb (clone AC 15, Sigma Aldrich, Taufkirchen, Germany, 1:5000). Proteins were visualized by the enhanced chemiluminescence system (GE Healthcare).

### 2.5. Real-Time Cell Analyzer

The cytotoxicity against PDAC cells was determined by a Real-Time Cell Analyzer (RTCA, X-Celligence, ACEA Biosciences, San Diego, CA, USA) in triplicates as described elsewhere [18,57]. Briefly, 5–10 × 10^3^ adherent PDAC cells/well in complete medium were added to 96-well micro-E-plate to monitor the impedance of the cells via electronic sensors (located at the bottom of the wells) every 5 min for up to ~24 h. The measured impedance of the PDAC cells is expressed as an arbitrary unit called cell index (CI), which reflects changes in cellular parameters such as cell proliferation, morphological changes (e.g., adherence, spreading) and cell lysis. The CI was normalized to 1 after PDAC cells reached their linear growth phase, since the initial adherence of PDAC cells in different wells can differ slightly. Thereafter, short-term activated Vγ9Vδ2 γδ T cells in 12.5 IU/mL rIL-2 at an E/T ratio of 25:1 were added as well as medium, previously titrated saturating concentrations of 300 nM PAg BrHPP or 1 µg/mL tribody [(HER2)_2_×Vγ9] or control constructs to the RTCA after ~24 h. For the determination of E/T ratio, cell numbers were counted shortly before the addition of appropriate cells to the RTCA assay.

When effector Vγ9Vδ2 γδ T cells induce lysis of the PDAC cells, the loss of impedance of tumor cells is shown as a decrease in the normalized CI. Additionally, as a positive control for complete lysis, PDAC cells were treated in several wells with a final concentration of 1% Triton X-100. For precise analysis of cytotoxicity, the cells were monitored every minute for the indicated time points. By using the RTCA software (ACEA Biosciences Inc.), the raw data files were exported to Microsoft Excel for further calculations, and then described as follows. The mean of Triton-X-100 samples was calculated and defined as 100% lysis 15 h after addition of γδ T cells. The ratio of each sample to spontaneous lysis of tumor cells alone was calculated and the ratio was normalized to maximal inducible lysis by Triton-X-100.

Experiments in which different PDAC cells were cocultured with short-term activated Vγ9Vδ2 T cells in medium or stimulated either by BrHPP or tribody [(HER2)_2_×Vγ9] were performed under equal conditions using different short-term activated Vγ9Vδ2 T cells of different donors in independent experiments.

### 2.6. Enzyme-Linked Immunosorbent Assay

To quantify IFN-γ, TNF-α or granzyme B released by T cells, 5 x 10^3^ PDAC cells in 100 µL of complete medium were cultured into 96-well microtiter plates (Nunc, Wiesbaden, Germany) for 24 h, and, thereafter, were cocultured with short-term activated Vγ9Vδ2 γδ T cells at an E/T ratio of 25:1 and 5:1 in the presence of medium or 1 µg/mL tribody [(HER2)_2_×Vγ9] together with 12.5 IU/mL rIL-2. After 6 or 24 h of coculture, supernatants were collected and stored at −20 °C until use. Human granzyme B was measured by a sandwich DuoSet ELISA kit (# DY1154 from R&D System), and human TNF-α and IFN-γ by human TNF-α or an IFN-γ DuoSet ELISA kit (both from R&D Systems), respectively, in duplicates, following the procedures outlined by the manufacturer.

### 2.7. CD107a-Degranulation Assay

In total, 5000 to 7500 PDAC cells in 50 µL complete medium in 96-well plates (Nunc) were cultured either with 50 µL of 1 mM NaOH solvent control, 1 mM 1-methyl-levo-trypthophan (1-l-MT, Sigma Aldrich) or 1 mM 1-methyl-dextro-tryptophan (1-d-MT, Sigma Aldrich), as shown in Figure 3, or with medium, as shown in Figure 5, overnight. After 24 h, short-term activated Vγ9Vδ2 γδ T cells with 12.5 IU/mL rIL-2 and 300 nM BrHPP were added at an E/T ratio of 25:1 to the indicated PDAC cells or cultured alone. In Figure 5, short-term activated Vγ9Vδ2 γδ T cells were cultured in medium, with 1 mM of previously titrated l-kynurenine (200 to 1000 µM, R&D System) or with 1 mM of previously titrated picolinic acid (Sigma Aldrich) for 24 h before coculturing these cells with different PDAC cells. For CD107a-assay, 10 µL FITC-labeled anti-human CD107a mAb clone H4A3 (50 µg/mL, BioLegend) was added directly, whereas 3 µM monensin (Merck) was added 1 h after coculturing the cells. After an additional 3 h, Vγ9Vδ2 γδ T cells were stained with PE-labeled anti-TCRγδ mAb (clone 11F2, BD Biosciences), and analyzed by flow cytometry.

### 2.8. Cell Cycle Analysis

Panc1 and PancTu-I cells were cultured at 5 × 10^5^ cells/well in 6-well plates in complete medium overnight. After 24 h, medium was removed, and cells were washed with serum-free medium. Thereafter, PDAC cells were cultured in serum-free medium without cytokines or with 10 ng/mL IFN-γ, 10 ng/mL TNF-α (both from R&D Systems) and the combination of both cytokines for 24 h. After incubation, supernatants were collected and PDAC cells were detached using 0.05% trypsin. Supernatant and detached cells were washed twice with cold PBS/5 mm EDTA and resuspended in 1 mL of PBS/5 mm EDTA. PDAC cells were fixed by adding 1 mL of ethanol and incubating for 30 min on ice. Thereafter, PDAC cells were centrifuged and resuspended in 0.5 mL of PBS/5 mm EDTA. RNA was removed by digestion with 4 μL of RNaseA (1 mg/mL) for 30 min at room temperature. After 1 h incubation with 0.5 mL of staining solution containing 50 μg/mL propidium iodide (PI) in PBS/5 mm EDTA, cell cycle analysis was performed by flow cytometry using a FACS Calibur Analyzer (BD Biosciences), and data were analyzed with FlowJo software.

### 2.9. Statistical Analysis

The statistical analysis was performed by using Graph Pad Prism (Graph Pad Software, Inc., La Jolla, CA, USA). The Shapiro–Wilk normality test was applied to analyze the normal distribution assumption. For parametric data of matched datasets, a paired, two-tailed t-test was used. All statistical tests were two sided and the level of significance was set at 5%. All appropriate tests were indicated in the figure legends.

## 3. Results

Since γδ T cell-based immunotherapy offers a promising approach, a profound understanding of the mediators, which influence T cell-mediated cytotoxicity, is required. Although γδ T cells can infiltrate into PDAC tissue and kill pancreatic tumor cells, they were not able to efficiently kill all tumors of PDAC patients, which seems to be due to different intrinsic resistance mechanisms. One of the intrinsic mechanisms could be the release of intermediates of the tryptophan pathway by PDAC cells. Different assays (FCM, WB, Real-Time Cell Analyzer, etc.) were used to investigate the cytotoxic capacity of γδ T cells against PDAC cells in the presence of either IDO inhibitors or IDO metabolites.

### 3.1. γδ T Cell Cytotoxicity against PDAC Cells with Different Susceptibility

**Figure 1:**
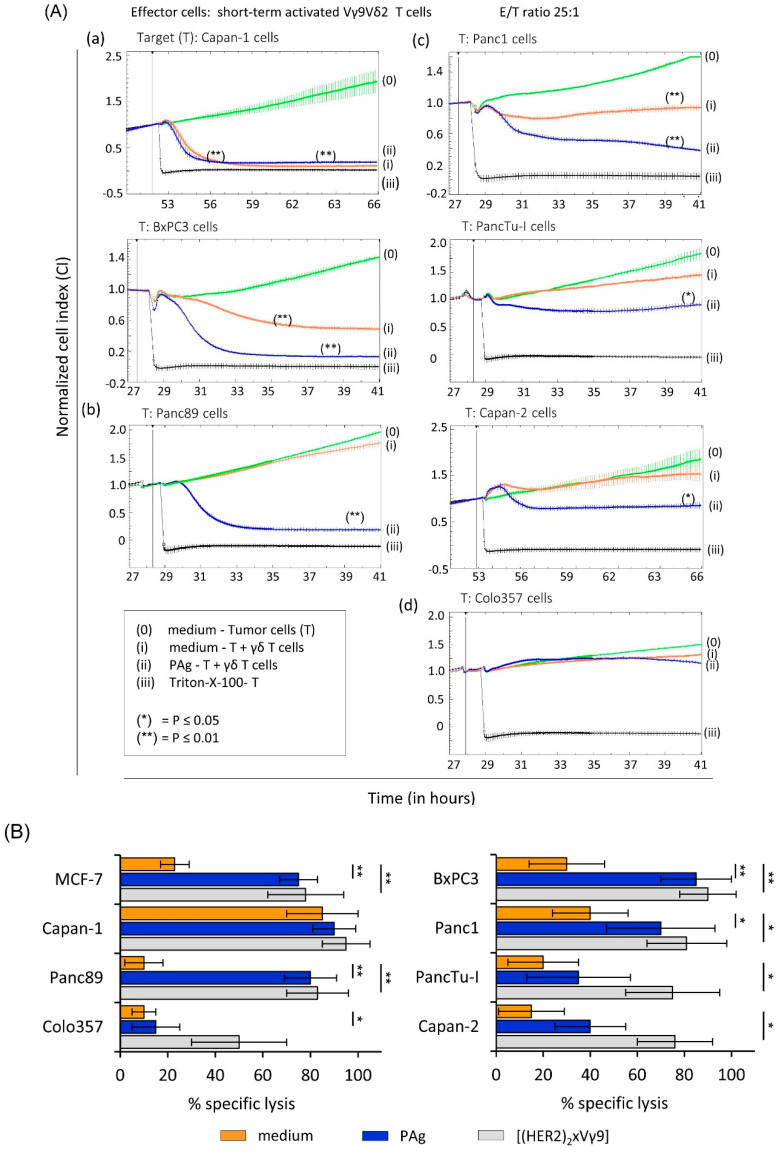
Variable efficacy of γδ T cell-mediated lysis against different pancreatic ductal adenocarcinoma (PDAC) cells. (**A**) Five to ten thousand tumor cells (T) of PDAC cells including (**a**) susceptible Capan-1 and BxPC3 cells, (**b**,**d**) resistant Panc89 and Colo357 cells and (**c**) almost resistant Panc1, PancTu-I and Capan-2 cells were cultured in complete medium measuring the impedance of the cells every three minutes using Real-Time Cell Analyzer (RTCA). Excluding tumor cells in medium alone [green, (0)] and the positive control for maximal lysis [Triton-X-100, black, (iii)], short-term activated Vγ9Vδ2 γδ T cells with 12.5 IU/mL rIL-2 were added after the initial growth phase of PDAC cells for 28 h at an E/T ratio of 25:1 to the tumor cells. γδ T cells were either stimulated with 300 nM PAg BrHPP [blue, (ii)] or cultured in medium [orange, (i)]. Presented are respresentative experiments with triplicate determinations +/− SD. (**B**) For validation, experiments were replicated with MCF-7, Capan-1, BxPC3, and Capan-2 cells (*n* = 3) and with Panc89, Colo357, Panc1 and PancTu-I cells (*n* = 10). Presented are the mean values of the different experiments (*n* = 3 to 10) with triplicate determinations +/− SD. The cytotoxic capacity of Vγ9Vδ2 γδ T cells against the indicated PDAC cells was calculated in the presence of medium (orange bars), 300 nM BrHPP (blue bars) or 1 μg/mL tribody [(HER2)_2_×Vγ9] (grey bars) in the presence of 12.5 IU/mL rIL-2 10 h after addition of effector cells as a % of specific lysis compared to control sample (without effector cells) and maximal lysis. Based on the assumption of normal distribution (Shapiro–Wilk normality test) of matched samples, statistical comparison was carried out parametrically by using paired, two-tailed t-test. Significances are shown as *p* values; * = *p* < 0.05; ** = *p* < 0.01.

In this study, we analyzed the susceptibility of several PDAC cells of different origin and stage of differentiation towards γδ T cell-mediated lysis. By using RTCA, we distinguished between three different sensitivities of PDAC cells against γδ T cell-mediated cytotoxicity as follows: (i) Capan-1 and BxPC3 cells were susceptible to the lysis mediated by short-term activated Vγ9Vδ2 T cells after restimulation of the cocultured γδ T cells with PAg BrHPP, while Capan-1 cells were also killed by Vγ9Vδ2 T cells in the absence of a further stimulus [Figure 1A (a)]; (ii) Panc89 cells were resistant to Vγ9Vδ2 T cell cytotoxicity but were killed in the presence of BrHPP [Figure 1A (b)]; (iii) Panc1, PancTu-I and Capan-2 cells were almost resistant but the resistance against Vγ9Vδ2 T cell cytotoxicity was partially abolished when Vγ9Vδ2 T cells were stimulated with BrHPP [Figure 1A (c)]; (iv) Colo357 cells were resistant against Vγ9Vδ2 T cell lysis of PDAC cells, also in the presence of BrHPP [Figure 1A (d)].

Taken together, we observed different levels of susceptibility of PDAC cells against Vγ9Vδ2 T cell-mediated lysis, which was modulated by restimulating short-term activated Vγ9Vδ2 γδ T cells with their selective antigens.

Recently, we demonstrated that tribody [(HER2)_2_×Vγ9] enhanced the Vγ9Vδ2 γδ T cell cytotoxicity against several PDAC cells such as Panc89 as well as PancTu-I cells, and partially against Colo357 cells [16]. The enhanced γδ T cell cytotoxicity, which was shown for resting as well as for short-term activated Vγ9Vδ2 T cells established from healthy donors or PDAC patients as well as for PDAC-infiltrating γδ T cells, was mainly mediated by the release of granzymes. In addition, we used other tribodies that have no specificity for γδ T cells or tumor cells as control constructs. Similar to our previous reports, the control constructs did not trigger target cell lysis ([16] and data not shown). Here, we observed that tribody [(HER2)_2_×Vγ9] significantly and more potently enhanced γδ T cell-mediated lysis of all PDAC cells in comparison to medium or BrHPP (Figure 1B).

Although the Vγ9Vδ2 γδ T cell-mediated lysis of PDAC cells in the presence of tribody [(HER2)_2_×Vγ9] revealed impressive results, substantial heterogeneity between the γδ T cell-mediated lysis of these different PDAC cells was observed when γδ T cells were not restimulated. To obtain more insights about tumor resistance against γδ T cell-mediated cytotoxicity, we examined some intrinsic tumor escape mechanisms.

### 3.2. Differential IDO Expression in PDAC Cells and Modulation of IDO-1 Expression by IFN-γ

**Figure 2:**
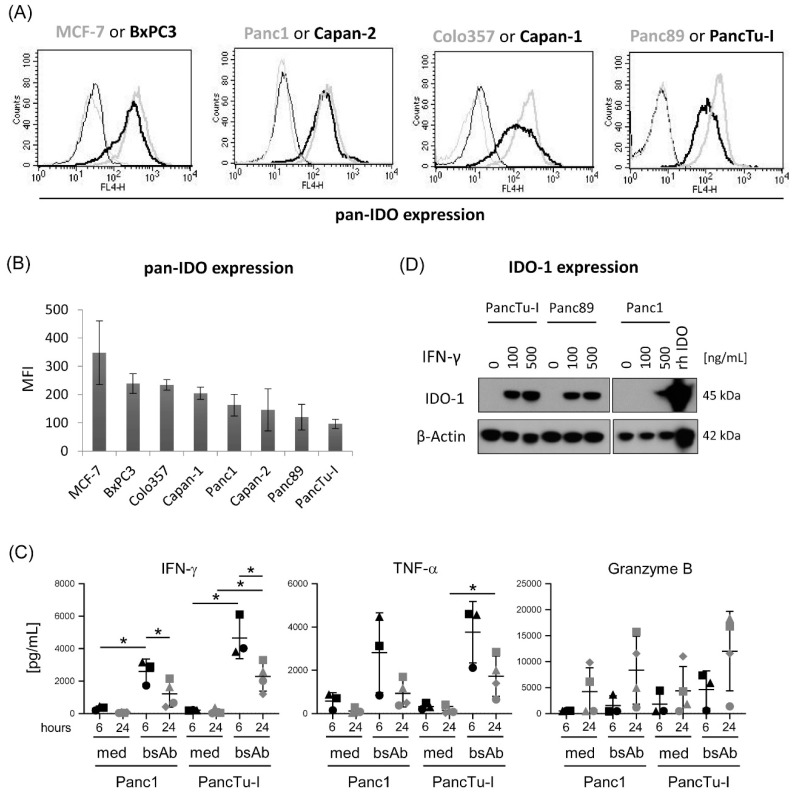
Intracellular expression of IDO in PDAC cells and release of γδ T cell mediators after coculture with PDAC cells. (**A**,**B**) Intracellular pan-IDO expression was analyzed by staining the indicated different PDAC cells with anti-pan-IDO-APC mAb (clone #700838) and by FACS Calibur Analyzer. (**a**) Histograms depicted are representative results of three different experiments. Thin black and grey lines represent isotype controls and thick black and grey lines represent the appropriate pan-IDO expression in the indicated PDAC cells. (**B**) Mean fluorescence intensity (MFI) of pan-IDO expression of three different experiments +/- SD is shown. (**C**) The 5–10 × 10^3^ Panc1 and PancTu-I cells were cultured in complete medium for 28 h in 96-well plates. Thereafter, PDAC cells were cocultured with short-term activated Vγ9Vδ2 γδ T cells of PDAC patients in 12.5 IU/mL rIL-2 at an E/T ratio of 25:1 in medium or stimulated with 1 μg/mL tribody [(HER2)_2_×Vγ9]. IFN-γ, TNF-α and granzyme B release was determined in the supernatant after 6 and 24 h using ELISA. Each symbol represents the data of one donor, and the lines represent the median values of different independent experiments. Significances are shown as *P* values; * = *p* < 0.05. (**D**) Indicated PDAC cells were left unstimulated or treated with 100 or 500 ng/mL IFN-γ for 48 h and were subsequently lysed. Protein was separated by SDS-PAGE, blotted on a nitrocellulose membrane and probed with anti-IDO1 mAb (clone 1A3) and detected by POD-conjugated sheep anti-mouse mAb. ß-actin mAb (clone AC 15) was used as loading control. Results of one out of two experiments are shown.

Recently, we demonstrated that the resistance of Colo357 cells to the cytotoxic activity of γδ T cells could be reversed by the combined usage of COX-2 inhibitors and [(HER2)_2_×Vγ9] [28]. Here, we analyzed whether other intrinsic tumor escape mechanisms such as overexpression of IDO, which can influence γδ T cell response, play a role. Regarding the intracellular pan-IDO expression, we observed that all analyzed PDAC cells expressed IDO but differed in their intensity of expression (Figure 2A,B). As a positive control for IDO overexpression, the breast cancer cell line MCF-7 was used. To distinguish between IDO-1 and IDO-2 expression, Western blot analysis with appropriate mAb was performed. A high expression of IDO-1 as well as of IDO-2 was observed in MCF-7, Colo357 and Capan-1 cells, while BxPC3 and Capan-1 also expressed both isoforms but with lower amounts. In contrast, PancTu-I, Panc89 and Panc1 cells lacked expression of IDO-1 but expressed a slight amount IDO-2 in comparison to IDO-1 (Figure S1).

Since IFN-γ is reported to enhance IDO-1 expression [58], we analyzed the release of IFN-γ by Vγ9Vδ2 γδ T cells in coculture with PDAC cells which are IDO-1 negative in the absence of cocultured γδ T cells, and a possible IFN-γ-induced modulation on the IDO-1 expression in these PDAC cells. We observed a very weak IFN-γ release of γδ T cells cocultured either with Panc1 or PancTu-I cells which could be significantly increased in the presence of tribody [(HER2)_2_×Vγ9] 6 h after coculture (Figure 2C, left panel). Interestingly, the treatment of PancTu-I and Panc89 cells with recombinant IFN-γ enhanced the IDO-1 protein expression in Panc89 and PancTu-I cells but not in Panc1 cells (Figure 2D). In addition, IDO-1 expression was not further increased after IFN-γ treatment in PDAC cells such as Colo357 cells where the expression was already very high (data not shown).

In parallel to IFN-γ release, we measured the release of TNF-α and granzyme B by γδ T cells, which can mediate cytotoxicity against PDAC cells. PancTu-I and Panc1 cells are almost resistant to CD95- or TRAIL receptor-induced cell death. Similar to IFN-γ, TNF-α was released by γδ T cells in coculture, with PDAC cells more intensive after 6 h compared to 24 h in the presence of tribody [(HER2)_2_×Vγ9], whereas granzyme B had its peak after 24 h of coculture (Figure 2C). While the effect of TNF-α on PDAC cells is described later in Figure 4, the degranulation of γδ T cells in coculture with PDAC cells and the effect of IDO inhibitors is reported in the following section.

### 3.3. Influence of IDO Inhibitors on the Interaction of PDAC Cells and γδ T Cells

IDO-1 and -2, as well as TDO, are key enzymes in tryptophan degradation, which catalyzes the reaction of tryptophan to N-formylkynurenine. In order to limit the availability of tryptophan for cancer cells and to enhance T cell immune responses, we tested four inhibitors in vitro as follows: (i) 1-methyl-l-tryptophan (1-l-MT, an IDO-1 inhibitor); (ii) 1-methyl-d-tryptophan (1-d-MT, an IDO-2 and TDO inhibitor); (iii) tenatoprazole (a proton pump inhibitor and IDO-2 inhibitor); (iv) 680C91 (a TDO inhibitor) [50,52,59,60]. We analyzed the effect of different concentrations (1 to 10,000 µM in logarithmic steps) of the inhibitors on T cells and PDAC cells, respectively, in order to exclude toxic effects potentially induced by these inhibitors. Concentrations of ≤ 1000 µM of 1-l-MT and 1-d-MT as well as the appropriate solvent control (NaOH) did not influence the cell growth of T cells and PDAC cells or the cell adherence of PDAC cells, whereas a concentration of 10,000 µM induced cell death in all cells (data not shown). Massive cell death was induced in T cells by treatment with tenatoprazole and 680C91 (both dissolved in DMSO), respectively, at concentrations higher than 1000 µM, and in PDAC cells with ≥ 100 µM. Additionally, lower concentrations of tenatoprazole and 680C91 but not of 1-l-MT and 1-d-MT negatively influence the adherence of PDAC cells which hamper the usage in the RTCA assay used for measurement of cytotoxicity (data not shown).

**Figure 3:**
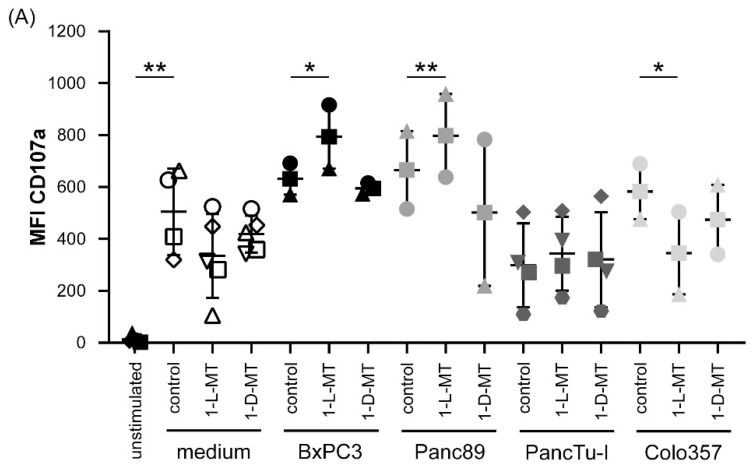

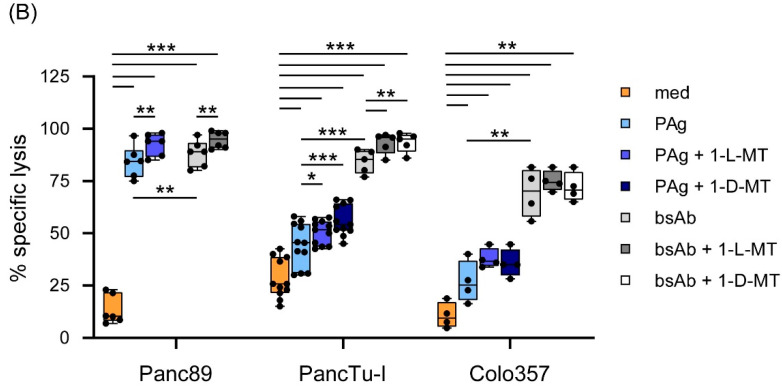
CD107 expression and cytotoxicity under the influence of IDO inhibitors. (**A**) In total, 5000 to 7500 indicated PDAC cells were cultured either in 1 mM NaOH solvent control (control) or with 1 mM 1-l-MT or 1 mM 1-d-MT for 24 h. Thereafter, short-term activated Vγ9Vδ2 γδ T cells with 12.5 IU/mL rIL-2 and 300 nM BrHPP were added at an E/T ratio of 25:1 to the indicated PDAC cells or cultured alone. As a control, γδ T cells with 12.5 IU/mL rIL-2 were left unstimulated. The CD107a expression (clone H4A3, 50 µg/mL) was measured after 4 h of culture by FACS Calibur Analyzer. Each symbol represents the data of one donor, and the lines represent the median values ± SD of different independent experiments. Based on the assumption of normal distribution (Shapiro–Wilk normality test) of matched samples, statistical comparison was carried out parametrically by using a paired, two-tailed t-test. Significances are shown as *p* values; * = *p* < 0.05; ** = *p* < 0.01. (**B**) In total, 5000 to 7500 indicated PDAC cells were cultured in 96-well E-plates for 28 h using RTCA. Short-term activated Vγ9Vδ2 γδ T cells with 12.5 IU/mL rIL-2 were added after the initial growth phase of PDAC cells at an E/T ratio of 25:1 to the PDAC cells. The γδ T cell cytotoxicity against the indicated PDAC cells was calculated in the presence of medium (orange bars), 300 nM BrHPP with medium (light blue bars), 1 mM 1-l-MT (middle blue bars) or 1 mM 1-d-MT (dark blue bars) and 1 μg/mL tribody [(HER2)_2_×Vγ9] with medium (light grey bars), 1 mM 1-l-MT (dark grey bars) or 1 mM 1-d-MT (white bars) 15 h after addition of effector cells as a % of specific lysis compared to the control sample (without effector cells) and maximal lysis. Experiments were replicated three to ten times under equal conditions using different short-term activated γδ T cells of different PDAC donors. Each symbol represents the data of one donor, and the lines represent the median values ± SD of different independent experiments. Based on the assumption of normal distribution (Shapiro–Wilk normality test) of matched samples, statistical comparison was carried out parametrically by using a paired, two-tailed t-test. Significances are shown as *p* values; * = *p* < 0.05; ** = *p* < 0.01; *** = *p* < 0.001.

Accordingly, we focused further experiments on the influence of 1-l-MT and 1-d-MT on the interaction of PDAC cells and T cells. Since the addition of tribody [(HER2)_2_×Vγ9] induced a significant increase in granzyme B release by short-term activated Vγ9Vδ2 γδ T cells cocultured with PDAC cells (Figure 2C), we investigated whether 1-l-MT or 1-d-MT enhance the degranulation of these γδ T cells after their restimulation in the presence or absence of the different PDAC cells. As a control, cells were cultured in the solvent control NaOH. Stimulation of short-term activated Vγ9Vδ2 γδ T cells increased their degranulation significantly, which was not significantly modulated by 1-l-MT and 1-d-MT, respectively. Interestingly, the degranulation of stimulated Vγ9Vδ2 γδ T cells was slightly increased in coculture with BxCP3 or Panc89 cells, which was further significantly enhanced in the presence of 1 mM 1-l-MT but not with 1 mM 1-d-MT (Figure 3A). In contrast, the coculture with PancTu-I, Colo357 and Panc1 cells did not influence the degranulation independently of the presence of these both inhibitors (Figure 3A, data not shown).

In parallel, we analyzed whether the γδ T cell-mediated cytotoxicity against PancTu-1 and Colo357 cells was influenced by 1-l-MT and 1-d-MT, although the degranulation of γδ T cells cocultured with these PDAC cells was not affected. As a positive control for the effect of 1-l-MT on cytotoxicity, we used the coculture of γδ T cells with Panc89 cells. We observed that 1-l-MT and 1-d-MT significantly enhanced the lysis of Panc89 and PancTu-I cells, but only slightly or not the lysis of Colo357 cells after coculturing them with γδ T cells stimulated with BrHPP or in the presence of tribody [(HER2)_2_×Vγ9], respectively (Figure 3B). While resistance of Colo357 cells against γδ T cell cytotoxicity is reported to be overcome by COX-2 inhibitors and tribody [(HER2)_2_×Vγ9] [28], resistance of PancTu-I cells can be completely overcome by IDO inhibitors plus tribody [(HER2)_2_×Vγ9] (Figure 3B). Tribody [(HER2)_2_×Vγ9] enhanced granzyme B release of γδ T cells cocultured with PancTu-I cells, but the addition of IDO-1 inhibitor 1-l-MT or 1-d-MT did not further enhance degranulation, suggesting that additional cytotoxic mediators play a role (Figure 2B and Figure 3B).

### 3.4. TNF-α Induced Cell Cyle Arrest in Panc1 Cells but Not in PancTu-I Cells

Since γδ T cells cocultured with either Panc1 or PancTu-I cells produced enhanced amounts of IFN-γ and TNF-α in the presence of tribody [(HER2)_2_×Vγ9] (Figure 2C) and both cytokines are described to induce cell cycle arrest, we examined the influence of both these cytokines on the proliferation of Panc1 and PancTu-I cells. To this end, either Panc1 or PancTu-I cells were cultured in medium or treated with 10 ng/mL each of IFN-γ and TNF-α or both cytokines for 24 h.

**Figure 4:**
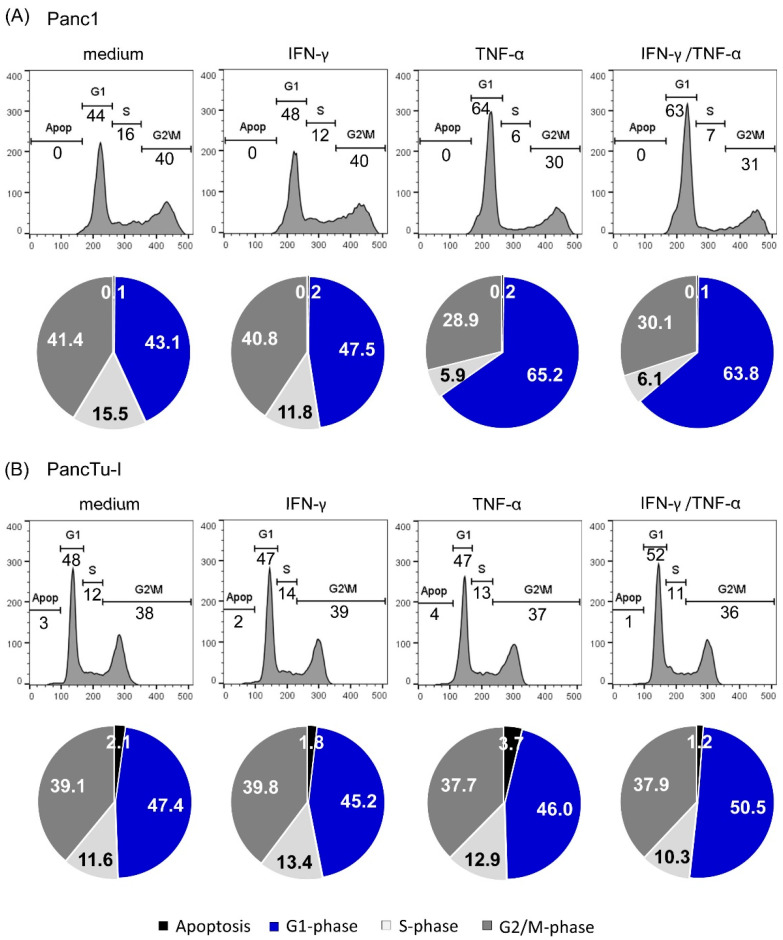
TNF-α-mediated G1 arrest in Panc1 cells. One hundred thousand (**A**) Panc1 cells or (**B**) PancTu-I cells were cultured in 6-well plates overnight. After 24 h, cells were left untreated (medium) or were treated with 10 ng/mL IFN-γ, 10 ng/mL TNF-α or 10 ng/mL IFN-γ together with TNF-α as indicated for 24 h. Cell cycle distribution of living cells obtained by gating in the forward/sideward scatter was determined using PI staining and flow cytometry. Numbers in the figures represent the percentage of the different cell cycle phases. One representative result is shown as a histogram (upper panel), and three independent experiments are represented as pie diagrams (lower panels). SD is < 10%.

Interestingly, when Panc1 cells were treated with TNF-α or with IFN-γ plus TNF-α, we observed a cell cycle arrest in the G1 phase in comparison to cells cultured in medium or treated with IFN-γ, suggesting that this arrest is mediated by TNF-α (Figure 4A). In contrast to Panc1 cells, treatment with cytokines did not significantly influence the cell cycle of PancTu-I cells, even though a very slight increase in G1 phase was detectable when PancTu-I cells were treated with both cytokines together (Figure 4B). In sum, TNF-α induced a cell cycle arrest in Panc1 cells but not in PancTu-I cells.

### 3.5. Treatment of γδ T Cells with Kynurenine Impaired γδ T Cell Cytotoxicity against PDAC Cells

**Figure 5:**
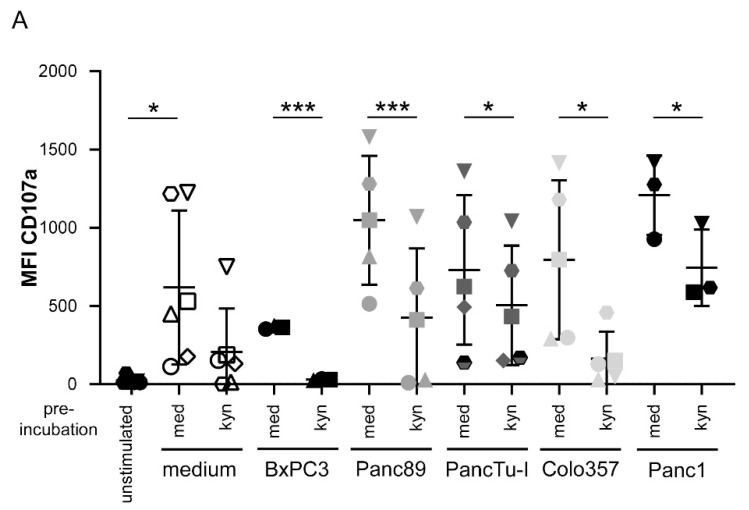

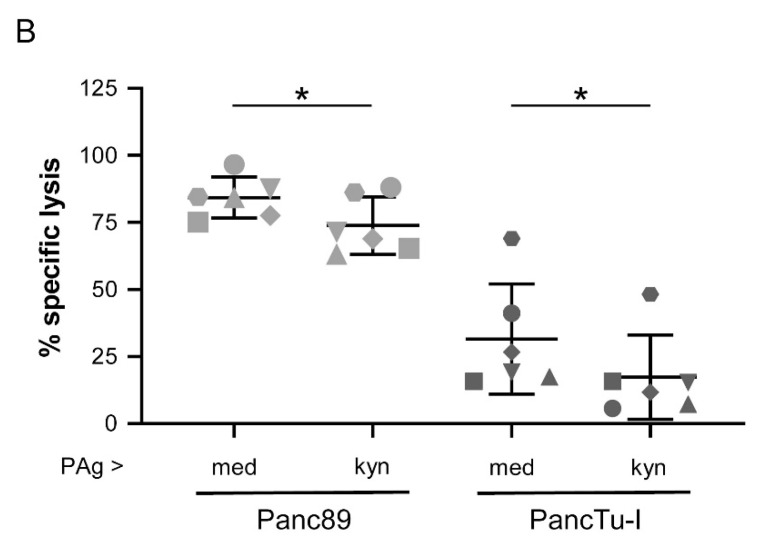
Decreased CD107a expression by kynurenine. (**A**) In total, 5000 to 7500 of the indicated PDAC cells were cultured in 96-well plates for 24 h. Simultaneously, short-term activated γδ T cells were cultured either in medium plus 12.5 IU/mL rIL-2 or, additionally, with 1 mM kynurenine. After incubation, γδ T cells were restimulated with 300 nM BrHPP and added to the PDAC cells at an E/T ratio of 25:1. CD107a (clone H4A3, 50 μg/mL) expression was measured after 4 h by FACS Calibur Analyzer using CellQuestPro software. Each symbol represents the data of one donor, and the lines represent the median value ± SD of different independent experiments. Based on the assumption of normal distribution (Shapiro–Wilk normality test) of matched samples, statistical comparison was carried out parametrically by using a paired, two-tailed t-test. Significances are shown as *p* values; * = *p* < 0.05; *** = *p* < 0.01. (**B**) Five thousand Panc89 and PancTu-I cells were cultured for 28 h using RTCA. Short-term activated Vγ9Vδ2 γδ T cells with 12.5 IU/mL rIL-2 and 300 nM PAg BrHPP were added after the initial growth phase of PDAC cells at an E/T ratio of 25:1 to the PDAC cells. The γδ T cell cytotoxicity against the indicated PDAC cells was calculated 15 h after addition of γδ T cells as a % of specific lysis compared to the control sample (without effector cells) and maximal lysis. Each symbol represents the data of one donor, and the lines represent the median values ± SD of different independent experiments. Based on the assumption of normal distribution (Shapiro–Wilk normality test) of matched samples, statistical comparison was carried out parametrically by using a paired, two-tailed t-test. Significances are shown as *p* values; * = *p* < 0.05.

In addition to PDAC cells, tumor-draining lymph nodes and mesenchymal stromal cells, endothelial cells as well as immune cells of the tumor microenvironment can overexpress IDO and release enhanced amounts of tryptophan metabolites such as kynurenine and picolinic acid [61,62,63]. Therefore, we examined the effects of recombinant kynurenine and picolinic acid on γδ T cell-mediated cytotoxic activity against PDAC cells. First, we observed that the treatment of activated γδ T cells with 1 mM kynurenine or 1 mM picolinic acid did not induce cell death after 24 h (data not shown), whereas kynurenine impaired the degranulation of activated γδ T cells (Figure 5). Additional coculture with the different indicated PDAC cells impaired significantly γδ T cell degranulation (Figure 5). Similar to the pretreatment of γδ T cells with kynurenine for 24 h but to a lower extent, the direct addition of kynurenine to γδ T cells cocultured with PDAC cells diminished γδ T cell degranulation (data not shown). In contrast, the treatment γδ T cells with picolinic acid did not reduce γδ T cell degranulation in presence or absence of PDAC cells (data not shown).

In line with the impaired γδ T cell degranulation, the addition of kynurenine to γδ T cells cocultured with PDAC cells diminished the PAg-stimulated γδ T cell cytotoxicity against PDAC cells (data not shown), interestingly, also significantly in Panc89 and PancTu-I cells, which express IDO-1 only after coculturing them with activated γδ T cells (Figure 3B). In sum, the data suggest that accumulation of kynurenine in the tumor microenvironment impaired the cytotoxic capacity of γδ T cells against PDAC cells.

## 4. Discussion

In this study, we confirmed that PDAC cells differ in their sensitivity against the cytotoxic activity of γδ T lymphocytes. With the exception of Capan-1 cells, all analyzed PDAC cells are largely resistant to the γδ T cell-mediated lysis unless they were restimulated. One mechanism of resistance towards killing by γδ T cells can be explained by an accumulation of the enzymes IDO-1 and IDO-2 in PDAC cells and its downstream molecule kynurenine which inhibits the degranulation and cytotoxicity of γδ T cells. In addition, treatment of γδ T cells with kynurenine drastically impaired the release of the granule content of γδ T cells independent of the coculture of PDAC cells. However, the interaction with PDAC cells further diminished the degranulation and cytotoxicity of γδ T cells. Interestingly, the cytotoxic activity of γδ T cells from different healthy donors and PDAC patients was very similar against the same PDAC cell line, suggesting that the heterogeneity of the PDAC cells has to be considered more closely.

The heterogeneity of pancreatic tumors is regarded as an obstacle for an effective immunotherapy for pancreatic cancer. The identification of individual diagnostic and prognostic markers within pancreatic cancer is therefore of great interest. IDO-1 is suggested as such a diagnostic marker of cancer therapy, especially since the tryptophan metabolic pathway is considered as a regulator of innate and adaptive immunity and has the ability to suppress T cell responses [51]. The overexpression and enhanced activity of IDO are described in both primary carcinogenic and metastatic tissues of different tumor entities and are always associated with poor clinical outcome in the majority of studies [32,39,41,42,43].

Prendergast and colleagues have reported that IDO is under the genetic control of the tumor suppressor *bridging integrator 1 (Bin1)*, which is attenuated in many human malignancies. The knockout of Bin1 in mouse model studies enhanced the STAT-1 and NF-κB-dependent expression of IDO [49,64]. While expression profile analysis of pancreatic endocrine tumors revealed an overexpression of *Bin1* gene [65], expression of Bin1 in PADC cells has not been analyzed so far.

In addition, a constitutive expression of IDO in human cancer is sustained by an autocrine signal mediated via STAT-3 activation by proinflammatory cytokine IL-6 and AHR activation by tryptophan catabolites such as kynurenine. IDO-mediated AHR activation, in turn, induced IL-6 expression [48]. In this context, it is of interest that the constitutive activation of inflamed STAT-3 and Janus kinase (Jak) signaling has been implicated in the development and progression of PDAC [66].

Here, we observed strong IDO-1 and -2 expression in PDAC cells of metastatic origin such as Colo357 and Capan-1 cells but not in Panc89 cells. In contrast, the pan-IDO expression in PDAC cells derived from primary tumors was weaker, with the exception of BxPC3 cells. This is quite in line with the reports of others who demonstrated that metastatic cancer cells in resected peri-pancreatic lymph nodes strongly overexpress IDO, whereas pancreatic cell lines derived from primary tumors did not unless they were treated with IFN-γ [37,54,55]. Witkiewicz et al. assumed that the influence of a cytokine-rich milieu (including IFN-γ) in the lymph nodes enhances IDO expression in metastatic pancreatic cancer cells which have traveled to the lymph nodes [54]. IFN-γ is involved in anti-viral and anti-tumor immune responses by triggering transcription of several genes such as *Ido-1* which is transcriptionally regulated by IFN-γ through activation of Jak-1 and STAT-1 but also NF-κB [67,68]. Although other soluble factors such as PGE_2_, vascular endothelial growth factor (VEGF) and IL-10 are described to play a role in the upregulation, IFN-γ is suggested to be the most important stimulus for IDO-1 expression [30]. Activation of IDO provides a negative feedback loop to control differentiation, activation and proliferation of T cells [69].

IDO overexpression by pancreatic tumor cells, other tumor cells and other immune cells was associated with the recruitment of Treg and the induction of tolerogenic T cell responses [54,70]. While the effect on γδ T cell effector function by IDO overexpressing tumor cells so far has not been examined, IDO is involved in the immunomodulatory capacities of human mesenchymal stem cells as well as of human mesenchymal stromal cells on γδ T cells, respectively. Fechter and colleagues demonstrated that IFN-γ released by activated γδ T cells induced a mesenchymal stem cell-mediated immunosuppression, which, in turn, exerted a negative feedback mechanism on cytokine production and proliferation of γδ T cells [71]. Additionally, cross-talk between mesenchymal stromal cells and anti-tumor reactive lymphocytes of the innate and adaptive arm of the immune system strongly drives tumor microenvironment to become immunosuppressive [62,63].

Interestingly, γδ T cells efficiently lysed Capan-1 cells overexpressing IDO, but not Panc89 and Colo357 cells. All three PADC cells were of metastatic origin. IDO inhibitors 1-l-MT and 1-d-MT enhanced γδ T cell cytotoxicity towards Panc89 cells, but not significantly against Colo357 cells. For Colo357 cells, we recently described an alternative tumor escape mechanism. TNF-α released by γδ T cells after their activation enhances the expression of COX-2 in Colo357 cells which increase the release of COX-2 metabolite PGE_2_ which, in turn, inhibits γδ T cell cytotoxicity [28]. A tumor-promoting role of COX-2 has been demonstrated in tumor-bearing mice studies, where deletion or selective inhibition of COX-2 decreased secretion of IL-10 and increased production of IL-12 and IFN-γ [72]. In addition, the ability of the selective COX-2 inhibitor celecoxib to prevent IDO activation and thereby enhancing an anti-tumor vaccination strategy has been reported in a mammary as well as in pancreatic carcinoma mouse model [73,74,75].

While γδ T cell-mediated cytotoxicity against Colo357 cells is almost complete in the presence of tribody [(HER2)_2_×Vγ9] administered together with COX-2 inhibitor DuP697 [28], the addition of IDO inhibitors 1-l-MT and 1-d-MT did not further enhance the cytotoxic activity of γδ T cells.

COX-2 has also been reported to induce IDO-1 through the release of PGE_2_ by human monocyte-derived dendritic cells (DCs). However, to fully activate IDO-1 in these DC, a second signal through TNF receptor or Toll-like receptors is required [76]. This is of relevance when a maturation cocktail including PGE_2_ was used for the generation of clinical-grade DC preparations injected in multiple myeloma (MM) patients. The application of these DCs correlated with a rapid expansion of Treg in a few MM patients enrolled in this clinical trial of anti-tumor DC-based vaccination [77].

Regarding PDAC cells established from primary tumor, all of them were almost resistant to γδ T cell cytotoxicity despite the low IDO-1 expression unless γδ T cells were restimulated by PAg or tribody [(HER2)_2_×Vγ9]. However, a weak IDO-2 expression in these PDAC cells might compensate for the absence of IDO-1 expression, because the addition of IDO-2 inhibitor 1-d-MT enhanced γδ T cell cytotoxicity against PancTu-I cells. Additionally, stimulated γδ T cells release IFN-γ after coculture with PDAC cells derived from primary tumors which modulate IDO-1 expression in these PDAC cells, since the application of IDO-1 inhibitor 1-l-MT increased the γδ T cell degranulation and cytotoxic activity against these PDAC cells. As a control, Panc1 cells served where IDO-1 was not expressed and not induced after treatment with IFN-γ. Interestingly, Panc1 cells were susceptible to TNF-α, which induced a cell cycle arrest in this PDAC cell line. Wieder and colleagues reported that T helper 1 cells producing TNF-α and IFN-γ can arrest the cancer cells in a permanently non-proliferating state called senescence [78]. While treatment of Panc1 cells with IFN-γ for 24 h did not induce cell cycle arrest, these cells were sensitive to TNF-α. However, prolonged culturing of Panc1 cells with TNF-α and IFN-γ over several passages drastically reduced the number of viable Panc1 cells (data not shown). Treatment of surviving cells with TNF-α but not with IFN-γ again induced cell cycle arrest, underlining the sensitivity of Panc1 cells to TNF-α treatment.

Since other stromal cells in the tumor microenvironment also overexpress IDO, we also analyzed the effect of IDO metabolite kynurenine on the γδ T cell cytotoxicity against PDAC cells. Treatment of activated γδ T cell with recombinant kynurenine reduced their degranulation already in the absence of PDAC cells. Impaired γδ T cell degranulation and cytotoxic activity were observed in the presence of PDAC cells after pretreatment of γδ T cells with recombinant kynurenine as well as after direct addition to the coculture, but the pretreatment was more intensive in comparison to the direct application. Kynurenine has been reported to accumulate in the tumor tissue of mice and humans [79,80]. Liu and colleagues measured up to 200 µM of kynurenine in the plasma of breast cancer patients [79]. In this study, we titrated kynurenine with 200 to 1000 µM and observed only a significant inhibition with the highest concentration of kynurenine, suggesting that the plasma kynurenine concentration of 200 µM reflects a high dilution of kynurenine compared to the suspected concentrations at the local immune cell–tumor cell interaction site in the tumor microenvironment.

Kynurenine was identified as an endogenous ligand of AHR [47]. Upon binding of kynurenine, AHR translocates into the nucleus, dimerizes with the AHR nuclear translocator, and induces the expression of its target genes by binding dioxin-responsive elements [81]. Sinclair and colleagues demonstrated that T cells have to be activated in order to induce the transport of kynurenine across the T cell membrane by the System L transporter SLC7A5 [82]. The activation of AHR has been reported to be implicated in the regulation of Treg and T helper 17 cells in mice as well as in exhaustion of T cells and influence on Treg and in humans [45,46,47,83]. Cibrian and colleagues demonstrated that the activation marker CD69 expressed by murine skin- and human-circulating γδ T cells is associated with the light chain glycoprotein-associated amino acid transporter complex LAT1 (CD98). CD69 regulates CD98 surface expression and uptake of L-tryptophan and thereby an AHR-dependent activation and IL-22 secretion in psoriatic mouse model as well as in psoriatic patients [84,85]. In this study, we demonstrated that kynurenine treatment of γδ T cells impaired degranulation and thereby their cytotoxic activity, which is of relevance for the anti-tumor response of γδ T cells.

Several IDO-1 inhibitors such as 1-MT (Indoximod), as well as Epacadostat, are already used or currently under investigation in several clinical trials (https://clinicaltrials.gov), with controversial results regarding the efficiency of IDO inhibitors [52,60,86,87]. Pharmacokinetic effects, the pretense of nutritional signals due to tryptophan mimicry and initiation of cellular detoxification affecting inflammatory pathways can prevent the efficiency of IDO inhibitors [60]. For instance, a phase I/II clinical trial named ECHO-203, which combined the application of Epacadostat together with programmed death-ligand (PD-L) 1 inhibitor Durvalumab, revealed no response in 15 patients with pancreatic cancer enrolled in this study [88]. Despite the negative findings of several studies, studies with IDO inhibitors including Epacadostat will continue. In addition, alternative approaches targeting downstream effectors of the IDO/TDO pathway are under investigation [51,53,60,88,89].

Of course, in addition to IDO overexpression, other inhibitory mechanisms including oxidative and metabolic stress and an immunosuppressive cytokine milieu in the tumor microenvironment as well as the expression of inhibitory receptors on tumors or tumor-surrounding cells are also important [7,8]. In addition, the differentiation of tumor-infiltrating Vγ9Vδ2 γδ T cells to ectonucleotidase CD73- or PD-L1 expressing immunosuppressive cells inhibiting T cell proliferation or the accumulation of senescent CD57-expressing TEMRA Vγ9Vδ2 γδ T cells after chemotherapy treatment of chronic myeloid leukemia patients did not improve anti-tumor function of these cells [90,91,92,93]. So far, it is uncertain whether in vivo application of tribody [(HER2)_2_×Vγ9] or similar bispecific antibodies in the presence or absence of IDO inhibitors or other inhibitors can overcome an immunosuppressive tumor microenvironment by targeting tumor-infiltrating γδ T cells to tumor cells. Therefore, a broader analysis of the resistance mechanisms of tumor cells towards γδ T cell-mediated cytotoxicity is of high interest. However, an adoptive transfer of ex vivo expanded allogeneic Vγ9Vδ2 γδ T cells together with tribody [(HER2)_2_×Vγ9] has been reported to reduce growth of pancreatic tumors engrafted into SCID/Beige immunocompromised mice [16].

## 5. Conclusions

Kynurenine which accumulates in IDO-overexpressing tumor cells or stromal cells of the tumor microenvironment impairs the degranulation and cytotoxic activity of γδ T cells, and thereby an effective anti-tumor response. Overexpression of IDO-1 and -2 in PDAC cells of metastatic origin as well as IDO-1 upregulation induced by IFN-γ released by γδ T cells after their activation and coculture with PDAC cells derived from primary tumors impaired γδ T cell degranulation and thereby their cytotoxic activity against these PDAC cells. Considering the heterogeneity of different PDAC escape mechanisms, the treatment with tribody [(HER2)_2_×Vγ9] drastically enhanced γδ T cell cytotoxicity against all PDAC cells, which can be further significantly enhanced by IDO inhibitors against several PDAC cells.

## Supplementary Materials

The following are available online at https://www.mdpi.com/2073-4409/9/5/1140/s1, Figure S1: Intracellular expression of IDO-1 and IDO-2 in different PDAC cell lines.
