# Supplementary material for: Influence of Indoleamine-2,3-Dioxygenase and Its Metabolite Kynurenine on γδ T Cell Cytotoxicity against Ductal Pancreatic Adenocarcinoma Cells
Source: Cells. 2020 May 6;9(5):1140. doi: 10.3390/cells9051140 (PMC7290398; doi:10.3390/cells9051140)
Supplement: Supplementary file 1 [file cells-09-01140-s001.pdf]

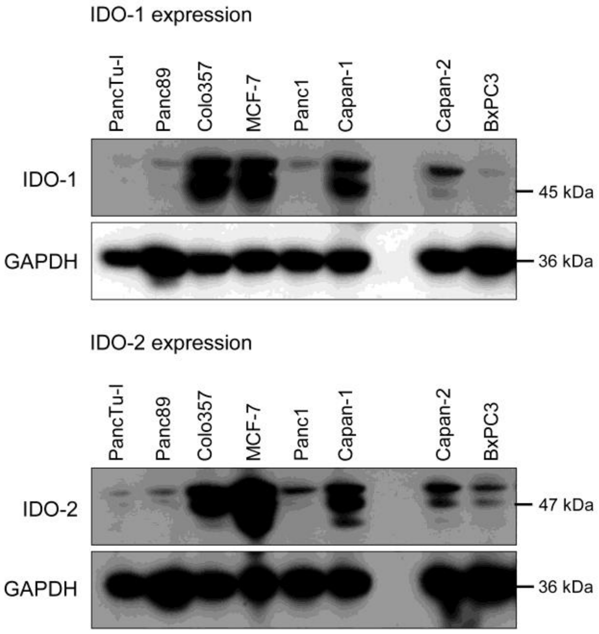

Supplemental Figure S1. Intracellular expression of IDO-1 and IDO-2 in different PDAC cell lines. Expression of IDO-1 and IDO-2 was detected by Western Blot. PDAC cells PancTu-I, Panc89, Colo357, Panc1, Capan-1 and -2, BxPC3 and breast cancer cells MCF-7 cells as positive control were lysed by TNE lysis buffer. 10 µg protein was separated by SDS-PAGE, blotted on a nitrocellulose membrane, probed with anti-IDO-1 mAb (clone 1A3, OriGeneTechnologies, 0.5 µg/ mL) or anti-IDO-2 mAb (clone 1A4, OriGeneTechnologies, 1 µg/ mL) and detected by POD conjugated sheep anti-mouse mAb (1:7500). For loading control blots were inactivated by 15% H_2O_2 and reprobed with anti-GAPDH mAb (clone 6C5, R&D Systems, BioTechne, 1:10000) detected by POD conjugated donkey anti-rabbit Ab. Results of one out of 2 experiments are shown.
